# 1966. Protection afforded by prior infection, vaccination, and hybrid immunity against symptomatic BA.1 and BA.2 Omicron infections.

**DOI:** 10.1093/ofid/ofac492.1591

**Published:** 2022-12-15

**Authors:** Heba N Altarawneh, Hiam Chemaitelly, Houssein H Ayoub, Patrick Tang, Mohammad R Hasan, Hadi M Yassine, Hebah A Al-Khatib, Maria K Smatti, Peter Coyle, Zaina Al-Kanaani, Einas Al-Kuwari, Andrew Jeremijenko, Anvar Hassan Kaleeckal, Ali Nizar Latif, Riyazuddin Mohammad Shaik, Hanan F Abdul-Rahim, Gheyath K Nasrallah, Mohamed Ghaith Al-Kuwari, Adeel A Butt, Hamad Eid Al-Romaihi, Mohamed H Al-Thani, Abdul Latif Al Khal, Roberto Bertollini, Laith J Abu-Raddad

**Affiliations:** Weill Cornell Medicine-Qatar, Doha, Ad Dawhah, Qatar; Weill Cornell Medicine - Qatar, Doha, Ad Dawhah, Qatar; Weill Cornell Medicine - Qatar, Doha, Ad Dawhah, Qatar; Sidra Medicine, Doha, Ad Dawhah, Qatar; Sidra Medicine, Doha, Ad Dawhah, Qatar; Qatar University, Doha, Ad Dawhah, Qatar; Qatar University, Doha, Ad Dawhah, Qatar; Qatar University, Doha, Ad Dawhah, Qatar; Hamad Medical Corporation, Doha, Ad Dawhah, Qatar; Hamad Medical Corporation, Doha, Ad Dawhah, Qatar; Hamad Medical Corporation, Doha, Ad Dawhah, Qatar; Hamad Medical Corporation, Doha, Ad Dawhah, Qatar; Hamad Medical Corporation, Doha, Ad Dawhah, Qatar; Hamad Medical Corporation, Doha, Ad Dawhah, Qatar; Hamad Medical Corporation, Doha, Ad Dawhah, Qatar; Qatar University, Doha, Ad Dawhah, Qatar; Qatar University, Doha, Ad Dawhah, Qatar; Primary Health Care Corporation, Doha, Ad Dawhah, Qatar; Hamad Medical Corporation, Doha, Ad Dawhah, Qatar; Ministry of Public Health, Doha, Ad Dawhah, Qatar; Ministry of Public Health, Doha, Ad Dawhah, Qatar; Hamad Medical Corporation, Doha, Ad Dawhah, Qatar; Ministry of Public Health, Doha, Ad Dawhah, Qatar; Weill Cornell Medicine, Doha, Ad Dawhah, Qatar

## Abstract

**Background:**

Protection offered by five different forms of immunity, combining natural and vaccine immunity, was investigated against symptomatic SARS-CoV-2 infection from Omicron BA.1 or BA.2, and severe, critical, or fatal COVID-19 from BA.1 or BA.2, in Qatar, between December 23, 2021 and February 21, 2022.

**Methods:**

Six national, matched, test-negative case-control studies were conducted on a sample of 272,861 PCR-positive tests and 669,628 PCR-negative tests to estimate effectiveness of BNT162b2 (Pfizer-BioNTech) vaccine, mRNA-1273 (Moderna) vaccine, natural immunity due to prior infection with pre-Omicron variants, and hybrid immunity from prior infection and vaccination.

**Results:**

Effectiveness of prior infection alone against symptomatic BA.2 infection was 46.1% (95% CI: 39.5-51.9%). Effectiveness of two-dose BNT162b2 vaccination alone was negligible at -1.1% (95% CI: -7.1-4.6), but nearly all individuals received their second dose >6 months earlier. Effectiveness of three-dose BNT162b2 vaccination alone was 52.2% (95% CI: 48.1-55.9%). Effectiveness of hybrid immunity of prior infection and two-dose BNT162b2 vaccination was 55.1% (95% CI: 50.9-58.9%). Effectiveness of hybrid immunity of prior infection and three-dose BNT162b2 vaccination was 77.3% (95% CI: 72.4-81.4%). Meanwhile, prior infection, BNT162b2 vaccination, and hybrid immunity all showed strong effectiveness ( >70%) against severe, critical, or fatal COVID-19 due to BA.2. Similar patterns of effectiveness were observed for BA.1 and for the mRNA-1273 vaccine.

**Figure 1.**
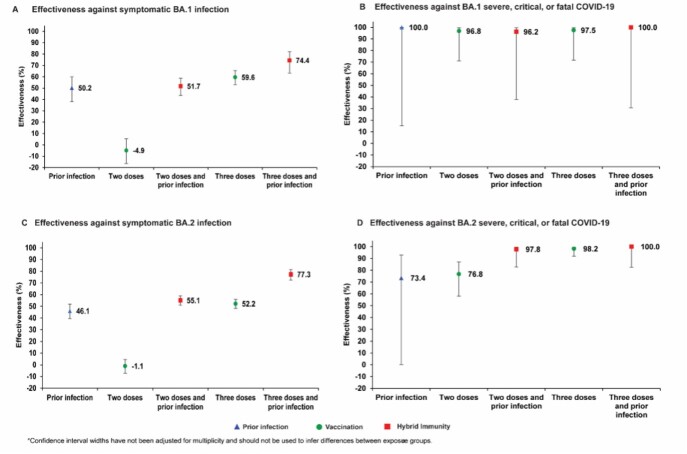
Effectiveness of prior infection, vaccination, and hybrid immunity against symptomatic Omicron infection and against severe, critical, or fatal COVID-19 for the BA.1 (panels A and B, respectively) and BA.2 (panels C and D, respectively) subvariants in the BNT162b2-vaccine study.

**Figure 2.**
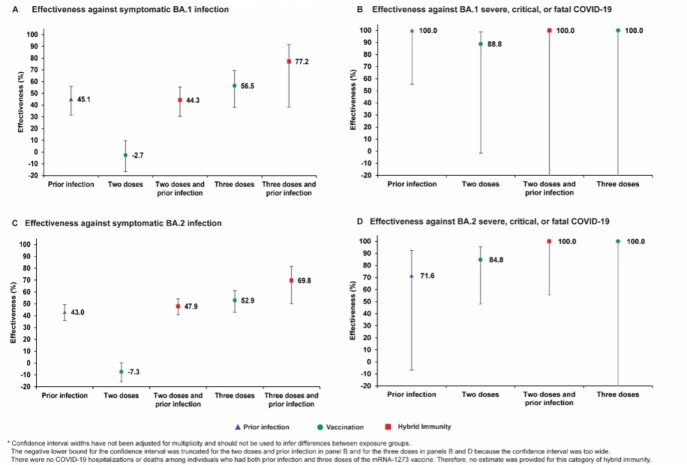
Effectiveness of prior infection, vaccination, and hybrid immunity against symptomatic Omicron infection and against severe, critical, or fatal COVID-19 for the BA.1 (panels A and B, respectively) and BA.2 (panels C and D, respectively) subvariants in the mRNA-1273-vaccine study.

**Conclusion:**

There are no discernable differences between BA.1 and BA.2 in the effects of prior infection, vaccination, and hybrid immunity. Vaccination enhances the protection of those with a prior infection. Hybrid immunity resulting from prior infection and recent booster vaccination conferred the strongest protection.

**Disclosures:**

**Adeel A. Butt, MBBS**, Gilead Sciences: Grant/Research Support.

